# Anxiety, depression, and fear after coronavirus disease 2019 infection and their association with long coronavirus disease symptoms

**DOI:** 10.3389/fpsyt.2025.1672447

**Published:** 2025-09-10

**Authors:** Ayaka Saito, Shiro Otake, Keiko Ohgino, Shogyoku Bun, Yu Mimura, Daisuke Ito, Naoki Miyazaki, Kengo Nagashima, Hideki Terai, Shotaro Chubachi, Katsunori Masaki, Jun Miyata, Ichiro Kawada, Ho Namkoong, Mizuha Hashiguchi, Junko Kagyo, Tetsuya Shiomi, Keita Masuzawa, Takanori Asakura, Sohei Nakayama, Yusuke Suzuki, Naoto Minematsu, Tadashi Manabe, Takahiro Fukui, Yohei Funatsu, Hidefumi Koh, Koichi Fukunaga

**Affiliations:** ^1^ Division of Pulmonary Medicine, Keio University School of Medicine, Tokyo, Japan; ^2^ Department of Neuropsychiatry, Keio University School of Medicine, Tokyo, Japan; ^3^ Department of Neurology, Keio University School of Medicine, Tokyo, Japan; ^4^ Memory Center, Keio University School of Medicine, Tokyo, Japan; ^5^ Biostatistics Unit, Clinical and Translational Research Center, Keio University Hospital, Tokyo, Japan; ^6^ Department of Infectious Diseases, Keio University School of Medicine, Tokyo, Japan; ^7^ Department of Internal Medicine, Keiyu Hospital, Kanagawa, Japan; ^8^ Department of Respiratory Medicine, Kitasato University Kitasato Institute Hospital, Tokyo, Japan; ^9^ Department of Clinical Medicine (Laboratory of Bioregulatory Medicine), Kitasato University School of Pharmacy, Tokyo, Japan; ^10^ Department of Internal Medicine, Hino Municipal Hospital, Tokyo, Japan; ^11^ Division of Pulmonary Medicine, Department of Internal Medicine, Tachikawa Hospital, Tachikawa, Japan

**Keywords:** anxiety, depression, fear, long COVID, mental health, symptoms

## Abstract

**Introduction:**

The coronavirus disease 2019 (COVID-19) pandemic has had widespread physical and psychological repercussions. Additionally, long COVID symptoms such as fatigue, dyspnea, and cognitive impairment have been well-documented; however, their associations with mental health symptoms remain unclear. This study investigated the relationships between long COVID and symptoms of anxiety, depression, and COVID-19-related fear using validated psychological assessment tools.

**Methods:**

This nationwide, prospective cohort study enrolled 1,066 individuals who recovered from COVID-19. The participants completed self-report questionnaires at 3, 6, and 12 months after diagnosis. Long COVID symptoms and psychological status were assessed using the Hospital Anxiety and Depression Scale (HADS) and Fear of COVID-19 Scale (FCV-19S). Statistical analyses were used to examine associations between long COVID symptoms and psychological scores while accounting for clinicodemographic factors.

**Results:**

Three months after diagnosis, 20.1% of the participants exhibited high anxiety (HADS-Anxiety [A] score ≥ 8), 23.6% had high depression (HADS-Depression [D] score ≥ 8), and 35.3% reported high levels of COVID-19-related fear (FCV-19S score ≥ 21). High HADS-A and HADS-D scores were significantly associated with younger age, female sex, and mild initial illness severity. Individuals with high HADS scores reported significantly greater long COVID symptoms; headaches and fatigue were associated with high anxiety scores and impaired concentration was associated with high depression scores.

**Conclusions:**

This study highlighted the significant associations between mental health symptoms and long COVID, emphasizing the need for integrated psychological support in post-COVID care. Addressing anxiety, depression, and fear-related concerns may contribute to improved management of long COVID symptoms and enhance overall patient well-being.

## 1 Introduction

Coronavirus disease 2019 (COVID-19) caused a global pandemic, which has led to severe health consequences for many individuals (1). After recovery from the acute infection phase, some patients experience persistent symptoms, collectively known as long COVID. The symptoms of long COVID include fatigue, breathing difficulties, impaired concentration, chest discomfort, joint pain, muscle pain, headaches, altered senses of smell and taste, sleep disturbances, fever, chills, sore throat, digestive complaints, and skin manifestations, which significantly diminish an individual’s quality of life (2).

In addition to its physical impacts, concerns have been raised regarding the potential mental health consequences of COVID-19 (3). Anxiety, fear, and social isolation related to physical illness can exacerbate psychological distress, leading to symptoms of anxiety and depression in many patients (4, 5). Moreover, these mental health challenges are associated with reduced labor productivity (6), highlighting their broader societal implications beyond individual well-being.

Long COVID is now increasingly recognized as a condition with both physical and psychological components. Persistent symptoms such as fatigue, dyspnea, and cognitive impairment may contribute to the development or worsening of anxiety, depression, and disease-specific fear. These domains are likely interrelated, yet their interactions have not been fully elucidated.

Current evidence addressing the relationship between physical manifestations of long COVID and mental health outcomes is limited. Few studies have examined how clinical and demographic factors influence susceptibility to psychological distress in this population (7, 8). Clarifying these associations is essential to identify at-risk groups, guide targeted interventions, and reduce the long-term burden of the pandemic.

Therefore, this study investigated the associations between long COVID and symptoms of anxiety, depression, and COVID-19-related fear and identified clinicodemographic factors associated with heightened susceptibility to these psychological symptoms. Enhancing our understanding of these relationships is essential for improving mental health assessments and facilitating effective support in clinical practice.

## 2 Materials and methods

### 2.1 Study design and participants

The detailed eligibility criteria for this study have been described previously (9). This prospective, nationwide cohort study enrolled participants aged 18 years and older. Eligible participants were admitted and discharged with a confirmed COVID-19 diagnosis based on positive polymerase chain reaction or antigen testing for severe acute respiratory syndrome coronavirus 2 (SARS-CoV-2) between January 2020 and February 2021 at 26 participating medical institutions.

Invitations for participation in this study were sent via mail to all eligible inpatients at the participating hospitals within a defined period. Patients who provided informed written consent were invited to complete a questionnaire either in paper format (paper patient-reported outcome [pPRO]) or via a smartphone application (electronic patient-reported outcome [ePRO]) at 3, 6, and 12 months after their diagnosis.

This study was approved by the Ethics Committee of Keio University School of Medicine (approval: #20200243). In addition, ethical approval and permissions to conduct the study were obtained from the institutional review boards of all the participating institutions.

### 2.2 Patient enrollment and questionnaire collection status

Overall, 2,923 study invitations were mailed from Keio University and 25 participating hospitals. Informed written consent was obtained from the 1,196 participants, and 1,066 valid medical records were collected from the electronic data capture system. Questionnaires were returned via pPRO or ePRO methods from 1,109, 1,034, and 840 patients at 3-, 6-, and 12-months post-diagnosis, respectively. Ultimately, 1,066 patients with complete medical information and 3-month post-diagnosis PRO data were included in the analyses.

### 2.3 Questionnaire

The following symptoms were identified after the COVID-19 diagnosis: fever (>37 °C); cough; sputum; breathlessness (dyspnea); hypersensitivity to sound, light, and smell (sensory hypersensitivity); weakness and fatigue (fatigue); hair loss (alopecia); joint pain (arthralgia); muscle pain (myalgia); difficulty with strength (muscle weakness); head pain (headache); sore throat; ringing in the ears (tinnitus); loss of consciousness (unconsciousness); abdominal pain; diarrhea; skin manifestations, such as bumps or redness (rash); numbness; eye-related symptoms (eye pain, itching, foreign body sensation, redness, watery eyes, and discharge); memory loss and difficulty finding words (memory impairment); and reduced ability to think and concentrate (poor concentration).

Participants were asked to indicate the presence of each symptom at the time of hospitalization and at 3, 6, and 12 months after diagnosis. Symptom duration was categorized into six time periods: <1 week, 1 week–1 month, 1–3 months, 3–6 months, 6–12 months, and >12 months. In this study, individuals who reported symptoms lasting at least 3 months from the initial onset were classified as having long COVID symptoms.

To investigate the mental health effects of COVID-19, we employed two validated instruments: the Hospital Anxiety and Depression Scale (HADS) and the Fear of COVID-19 Scale (FCV-19S). The HADS is a widely used tool for quantitatively assessing anxiety and depression (10), with proven validity and reliability in Japanese populations (11). The FCV-19S, a brief and easy-to-administer measure, specifically quantifies fear related to COVID-19 and is suitable for both research and clinical use (12). Participants completed questionnaires incorporating these scales, along with other internationally validated measures of mental health-related quality of life. A HADS-Depression [D] score ≥ 8 was classified as high depression, HADS-Anxiety [A] score ≥ 8 as high anxiety (13), and FCV-19S score ≥ 21 as high fear (12).

### 2.4 Statistical analyses

The baseline characteristics were summarized using the median and interquartile range for continuous variables and counts with proportions for categorical variables. Continuous variables were compared using Student’s t-test, whereas categorical variables were analyzed using the chi-squared test.

For the longitudinal analyses, a generalized mixed-effects model for repeated measures was employed, utilizing a logit link function and assuming an unstructured covariance matrix for each visit. This approach evaluated the impact of the post-acute sequelae and patient characteristics on anxiety and depression while accounting for missing data. If model convergence was not achieved, the covariance structure was adapted sequentially to Toeplitz, heterogeneous compound symmetry, first-order autoregressive, compound symmetry, and variance component until convergence was reached. The model included time, age, sex, smoking, oxygen requirement, comorbidity, use of antiviral drugs, education, use of steroids, fever, cough, sputum, dyspnea, sensory hypersensitivity, fatigue, alopecia, arthralgia, myalgia, muscle weakness, headache, sore throat, tinnitus, unconsciousness, abdominal pain, rash, numbness, eye-related symptoms, memory impairment, poor concentration, sleeping disorders, taste disorders, olfactory disorders, and diarrhea, and interaction terms between time and other variables as fixed effects. Adjusted odd ratios (ORs) and their 95% confidence intervals (CIs) for associations with the outcomes over time were estimated.

All statistical analyses were performed using SPSS version 26 (IBM Corp., Armonk, NY, USA) and SAS version 9.4 (SAS Institute, Cary, NC, USA). P-values < 0.05 were considered significant.

## 3 Results

### 3.1 Participant characteristics

The participant characteristics are summarized in **Supplementary Table S1**. A total of 1,066 individuals who recovered from COVID-19 participated in this study. The participant mean age was 56 years; 387 were women (36.3%) and 679 were men (63.7%). Overall, 374 (36.8%) participants reported a history of smoking.

The most commonly observed comorbidities were hypertension (344 participants, 32.3%), diabetes mellitus (178 participants, 16.7%), chronic kidney disease (46 participants, 4.3%), and bronchial asthma (55 participants, 5.2%). Overall, 343 participants (32.2%) had severe COVID-19 requiring oxygen supplementation. Additionally, 328 participants (30.8%) received systemic corticosteroid therapy, and 358 (33.3%) were treated with antiviral agents.

### 3.2 HADS scores

**Figure 1:**
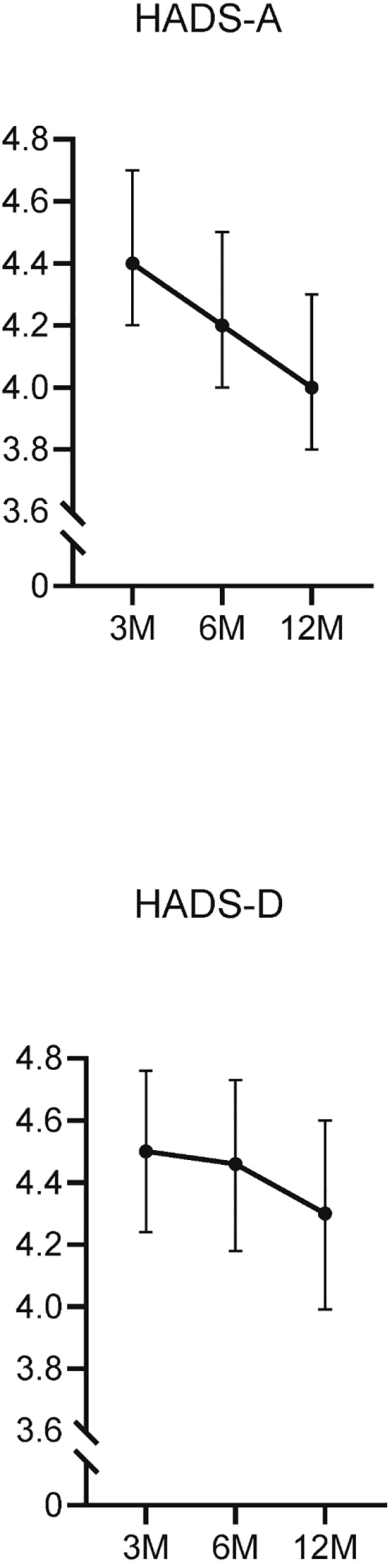
Longitudinal changes in the HADS-A and HADS-D scores. This figure illustrates the changes in Hospital Anxiety and Depression Scale-Anxiety (HADS-A) and Depression (HADS-D) scores over time, measured at 3, 6, and 12 months post-diagnosis.

Three months after diagnosis, 203 participants (20.1%) exhibited high HADS-A scores (≥8) (**Supplementary Table S2**). The mean HADS-A and HADS-D scores were 4.4 ± 3.9 and 4.5 ± 4.2, respectively. The mean HADS-A scores underwent a gradual decline at 3, 6, and 12 months (4.4, 4.2, 4.0) (**Figure 1**). Participants with high HADS-A scores were generally younger and had lower rates of oxygen supplementation, corticosteroid usage, and antiviral therapy across all time points. At 12 months, this group also exhibited a lower prevalence of hypertension (29.2% vs. 37.8%) and diabetes (12.3% vs. 18.5%).

Similarly, at 3 months, 236 participants (23.6%) had high HADS-D scores (≥8) (**Supplementary Table S3**). The mean HADS-D scores also showed a gradual decrease over time (4.5, 4.4, 4.3) (**Figure 1**). At 6 months, participants with high HADS-D scores were younger (54.3 ± 18.0 vs. 57.1 ± 16.0), had a lower body mass index (BMI) (23.8 ± 4.7 vs. 24.4 ± 4.0), and lower rates of oxygen supplementation(26.9% vs. 33.4%). Furthermore, the frequency of corticosteroid and antiviral therapy use was lower in this group at 6 and 12 months.

### 3.3 FCV-19S and its association with HADS scores

The mean FCV-19S score was 17.9 ± 5.9. The FCV-19S scores were elevated (≥21) in 35.3% of participants, with no discernible downward trend observed at 3, 6, or 12 months (17.9, 17.5, 17.4) (**Supplementary Figure S1**). Participants with high FCV-19S scores were generally older, frequently women, and underwent oxygen supplementation, corticosteroid administration, and antiviral agent administration at higher rates. These participants also exhibited a greater prevalence of comorbidities, including hypertension, diabetes, and hyperuricemia (**Supplementary Table S4**).

Although the characteristics of the participants with high FCV-19S scores differed from those with high HADS-A or HADS-D scores, participants with high HADS-A and HADS-D scores exhibited elevated FCV-19S scores. Moderate correlation was identified between HADS-A, and FCV-19S scores (correlation coefficient = 0.45, p < 0.0001), and similarly between HADS-D and FCV-19S (correlation coefficient = 0.38, p < 0.0001) (**Supplementary Figure S2**).

### 3.4 Association between HADS scores and long COVID

**Figure 2:**
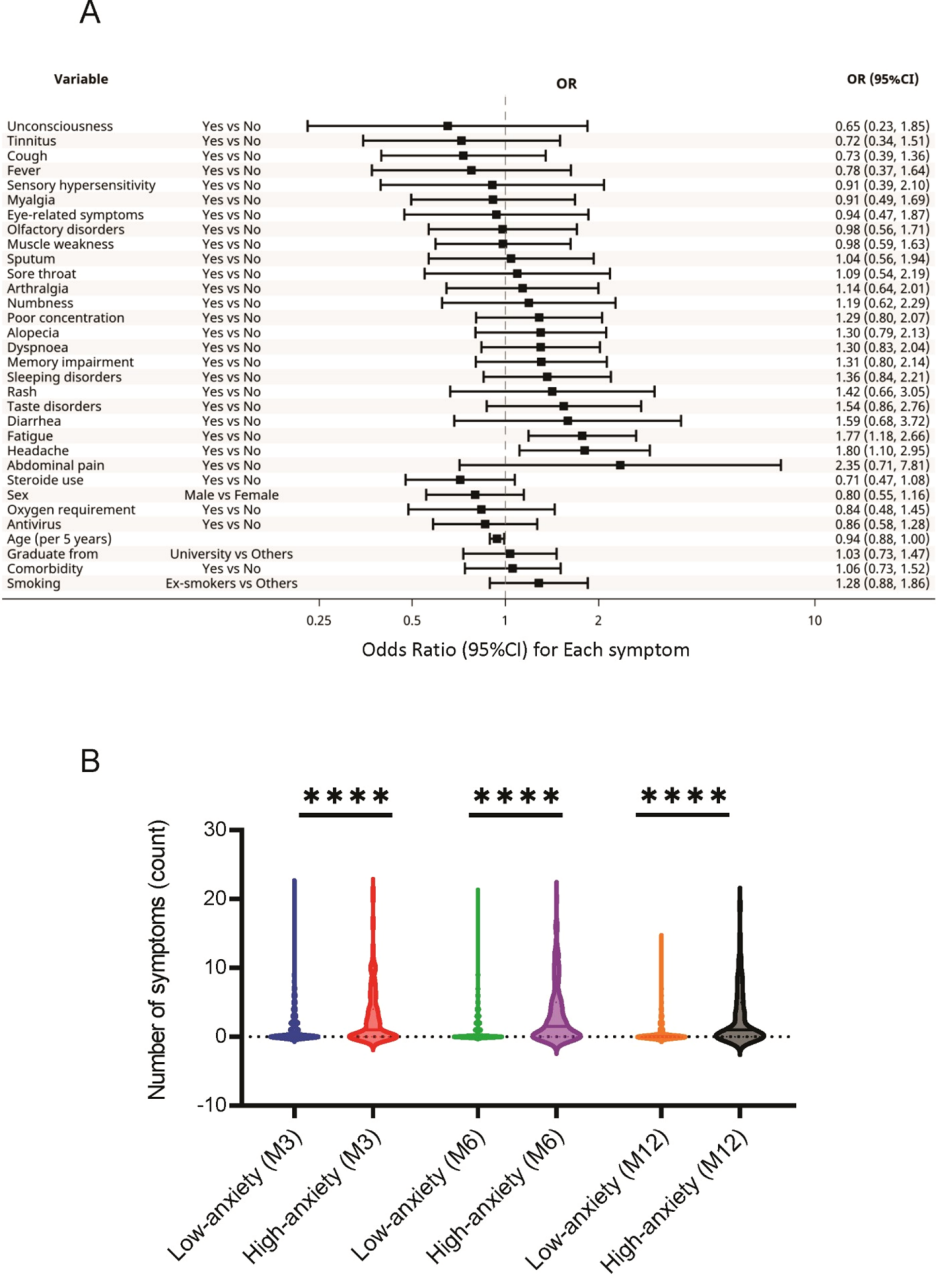
Association between HADS-A and long COVID symptoms. **(A)** Specific long COVID symptoms associated with high HADS-A scores. **(B)** Relationship between the HADS-A scores and the number of long COVID symptoms reported by the participants. **** P < 0.0001. COVID, coronavirus disease; HADS-A, Hospital Anxiety and Depression Scale-Anxiety.

The analysis across 3, 6, and 12 months revealed that the characteristics and long COVID symptoms associated with high HADS-A scores included younger age (OR, 0.94; 95% CI, 0.88-1.00), headache (OR, 1.80; 95%CI, 1.10-2.95), and fatigue (OR, 1.77; 95%CI, 1.18-2.66) (**Figure 2**). Interestingly, factors such as age, oxygen supplementation, corticosteroid use, and antiviral agent use, which differed significantly between the high and low HADS-A groups (**Supplementary Table S2**), were not associated with high HADS-A scores in this analysis.

**Figure 3:**
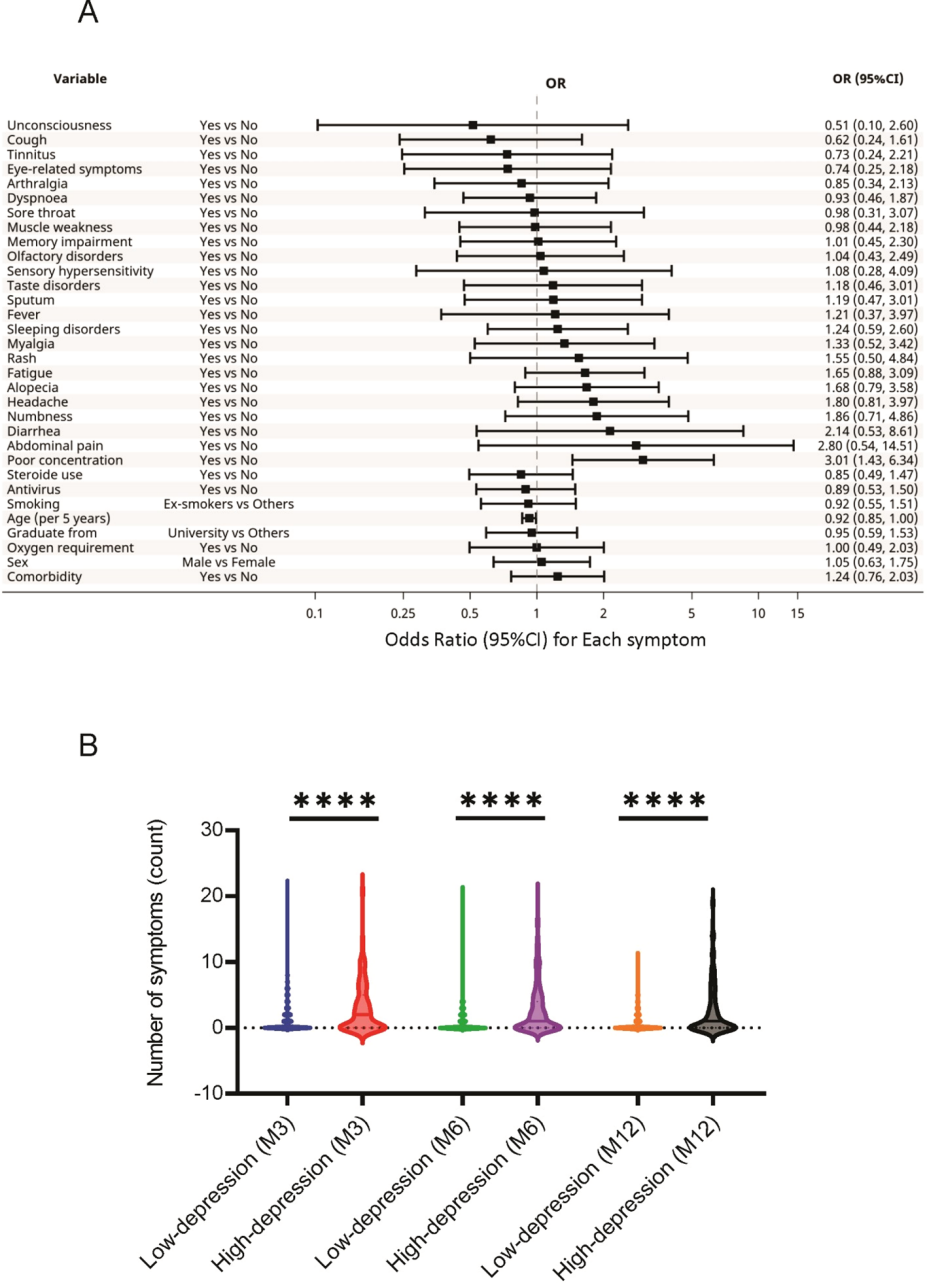
Association between the HADS-D and long COVID symptoms. **(A)** Specific long COVID symptoms associated with high HADS-D scores. **(B)** Relationship between HADS-D scores and the number of long COVID symptoms reported by the participants. **** P < 0.0001. COVID, coronavirus disease; HADS-D, Hospital Anxiety and Depression Scale-Depression.

For HADS-D, high scores were associated with younger age (OR, 0.92; 95%CI, 0.85-1.00) and impaired concentration (OR, 3.01; 95%CI, 1.43-6.34) (**Figure 3**). Similarly, factors such as BMI, oxygen supplementation, corticosteroid use, and antiviral agent use, which differed significantly between the high and low HADS-D groups (**Supplementary Table S3**), were not associated with high HADS-D scores in this analysis.

The relationships between the 24 sequelae symptoms and HADS scores were also examined. The participants with higher HADS-A scores reported significantly greater long COVID symptoms at 3, 6, and 12 months than those with low scores (**Figure 2**). A similar trend was observed in participants with high HADS-D scores (**Figure 3**).

High FCV-19S scores were associated with female sex (OR, 0.52; 95%CI, 0.31-0.89) and headache (OR, 3.45; 95%CI, 1.31-9.09) (**Supplementary Figure S3**). Additionally, participants with high FCV-19S scores reported significantly greater long COVID symptoms at 3, 6, and 12 months than those with low scores (**Supplementary Figure S3**).

## 4 Discussion

This study highlighted the association between mental health, as assessed by the HADS, and the prevalence of post-COVID symptoms by conducting a longitudinal evaluation of individuals recovering from COVID-19. Notably, this was the first study to demonstrate that individuals with high HADS-A and HADS-D scores consistently experienced multiple post-COVID symptoms over time. Furthermore, these individuals tended to exhibit elevated scores on the FCV-19S, a tool specifically designed to measure fear related to COVID-19 infection. These findings underscore the significant relationship between mental health and post-COVID symptoms, which was quantified using validated indices. Although there are several previous studies showing an association between long COVID symptoms and mental health (14–17), this study conducted a longitudinal analysis over 12 months and used multiple assessment tools to robustly clarify this association.

The participants with high HADS-A and HADS-D scores shared similar characteristics, including younger age, female sex, and mild severity of illness. Previous studies have reported that younger individuals are more likely to exhibit elevated HADS scores (18), potentially due to heightened vulnerability to social stressors and uncertainties about the future (19). Similarly, women are more prone to higher HADS scores than men (20), possibly due to factors such as life events (pregnancy and childbirth), hormonal fluctuations, and exposure to social stressors at home or work (21). The stress and anxiety induced by COVID-19 may have exacerbated these pre-existing vulnerabilities, particularly in younger individuals and women.

The elevated HADS scores in individuals with mild COVID-19 suggest that even those with less severe illness may experience significant mental health challenges. Younger individuals, who are more frequently represented among mild cases, may contribute to this trend. These findings emphasize the importance of addressing the mental health of individuals with mild COVID-19, a group that is often overlooked in clinical practice. Similar trends have been noted in previous studies, which linked younger age and female sex to higher levels of anxiety and depression (14) and reported a greater prevalence of anxiety and depression in mild cases compared with severe cases requiring hospitalization (22).

The participants with high HADS-A and HADS-D groups also exhibited elevated FCV-19S scores. As mentioned earlier, the FCV-19S provides a quantitative assessment of COVID-19-specific fears. This suggests that individuals experiencing general anxiety and depressive symptoms may be more susceptible to an intensified fear of COVID-19 infection, potentially exacerbating their psychological distress. Conversely, the heightened fear and uncertainty surrounding COVID-19 may act as a psychological stressor, contributing to the onset or worsening of general anxiety and depressive symptoms. Although prior studies have evaluated HADS-A and HADS-D in individuals with COVID-19 (23–25), only few have explored the multifaceted relationships among mental health symptoms, fear of COVID-19, and post-COVID symptoms (7, 17). This study is significant because it assessed anxiety and depressive symptoms and fear of COVID-19 over time, examined their interrelationships, and evaluated their association with post-COVID sequelae.

The findings of the present study indicated that individuals with high HADS-A and HADS-D scores were not only prone to vague feelings of insecurity and depression but also experienced specific fears and anxieties about COVID-19. This underscores the need for educational interventions targeting individuals with persistent mental health symptoms after COVID. Previous studies have shown that patient education improves the quality of life of COVID-19 survivors (26), suggesting the potential benefit of providing accurate information about COVID-19 to mitigate psychological distress.

In the analysis of the relationship between mental health and long COVID symptoms, individuals with high HADS-A and HADS-D scores reported a significantly greater number of symptoms. Specifically, high HADS-A scores were associated with headaches and fatigue, whereas high HADS-D scores were linked to impaired concentration. Headaches are frequently triggered by stress and anxiety, possibly due to sympathetic nervous system imbalances or muscle tension induced by stress responses (27). Impaired concentration is a hallmark symptom of depression. These findings suggest a strong relationship between mood and anxiety symptoms and long COVID. The role of stress in exacerbating physical symptoms has been well-documented (28), emphasizing the importance of addressing mental health complementary to direct treatment of post-COVID symptoms. However, individuals with certain physical illnesses or symptoms have a higher incidence of mental disorders (29), suggesting that these physical conditions may contribute to depression onset.

This study had a few limitations. First, the baseline mental health status prior to COVID-19 was not recorded, encumbering assessment of the extent of changes in HADS scores compared to pre-COVID levels. As a result, the causal relationship between COVID-19 infection, post-COVID conditions, and mental health symptoms remains uncertain. It cannot be ruled out that this only reflects the mental state prior to COVID-19 infection, and the lack of such data is a major limitation. Second, the study focused on individuals infected with the Delta variant of SARS-CoV-2 between January 2020 and February 2021. Future analyses of other variants, such as Omicron BA.1, BA.2, and BA.5, would strengthen the generalizability of the findings.

In conclusion, this study underscores the critical importance of understanding the mental health of individuals with post-COVID conditions. Recognizing the psychological needs of these individuals and providing comprehensive support and education may improve the management of post-COVID conditions. Specifically, in clinical practice, it may be useful to provide early intervention by psychiatrists and psychologists to patients with multiple long COVID symptoms. Future research is warranted to determine whether psychological interventions can directly alleviate long COVID symptoms.

## Data Availability

The datasets presented in this article are not readily available due to ethical restrictions. Requests to access the datasets should be directed to the corresponding author.

## References

[1] Chow EJ Uyeki TM Chu HY. The effects of the COVID-19 pandemic on community respiratory virus activity. Nat Rev Microbiol. (2022) 21:195–210. doi: 10.1038/s41579-022-00807-9, PMID: 36253478 PMC9574826

[2] Davis HE McCorkell L Vogel JM Topol EJ. Author correction: long COVID: major findings, mechanisms and recommendations. Nat Rev Microbiol. (2023) 21:133–46. doi: 10.1038/s41579-023-00896-0, PMID: 36639608 PMC9839201

[3] Zawilska JB Kuczyńska K. Psychiatric and neurological complications of long COVID. J Psychiatr Res. (2022) 156:349–60. doi: 10.1016/j.jpsychires.2022.10.045, PMID: 36326545 PMC9582925

[4] Zalai D Szeifert L Novak M. Psychological distress and depression in patients with chronic kidney disease. Semin Dial. (2012) 25:428–38. doi: 10.1111/j.1525-139X.2012.01100.x, PMID: 22809005

[5] Zawada K Bratek A Krysta K. Psychological distress and social factors in patients with asthma and chronic obstructive lung disease. Psychiatr Danub. (2015) 27:S462–4., PMID: 26417817

[6] Shigematsu L Kimura R Terai H Mimura Y Ito D Bun S. Social impact of brain fog and analysis of risk factors: long COVID in Japanese population. Ann Clin Transl Neurol. (2024) 11:2188–200. doi: 10.1002/acn3.52139, PMID: 38961833 PMC11330230

[7] Hoşgören Alıcı Y Çınar G Hasanlı J Ceran S Onar D Gülten E. Factors associated with progression of depression, anxiety, and stress-related symptoms in outpatients and inpatients with COVID-19: a longitudinal study. Psych J. (2022) 11:550–9. doi: 10.1002/pchj.557, PMID: 35593144 PMC9347536

[8] Zalaquett N Lutchman K Iliaki E Buley J Nathan N Sotos Prieto M. Findings associated with prolonged COVID-19 recovery among Boston healthcare workers. J Occup Environ Med. (2024) 66:962–9. doi: 10.1097/JOM.0000000000003221, PMID: 39196796

[9] Nakagawara K Morita A Namkoong H Terai H Chubachi S Asakura T. Longitudinal long COVID symptoms in Japanese patients after COVID-19 vaccinations. Vaccine X. (2023) 15:100381. doi: 10.1016/j.jvacx.2023.100381, PMID: 37731516 PMC10507639

[10] Zigmond AS Snaith RP. The hospital anxiety and depression scale. Acta Psychiatr Scand. (1983) 67:361–70. doi: 10.1111/j.1600-0447.1983.tb09716.x, PMID: 6880820

[11] Matsudaira T Igarashi H Kikuchi H Kano R Mitoma H Ohuchi K. Factor structure of the Hospital Anxiety and Depression Scale in Japanese psychiatric outpatient and student populations. Health Qual Life Outcomes. (2009) 7:42. doi: 10.1186/1477-7525-7-42, PMID: 19445722 PMC2687424

[12] Midorikawa H Tachikawa H Aiba M Shiratori Y Sugawara D Kawakami N. Proposed cut-off score for the Japanese version of the fear of coronavirus disease 2019 scale (FCV-19S): evidence from a large-scale national survey in Japan. Int J Environ Res Public Health. (2022) 20:429. doi: 10.3390/ijerph20010429, PMID: 36612751 PMC9819218

[13] Singer S Kuhnt S Götze H Hauss J Hinz A Liebmann A. Hospital anxiety and depression scale cutoff scores for cancer patients in acute care. Br J Cancer. (2009) 100:908–12. doi: 10.1038/sj.bjc.6604952, PMID: 19240713 PMC2661775

[14] Bota AV Bratosin F Bogdan I Septimiu-Radu S Ilie AC Burtic SR. Assessing the quality of life, coping strategies, anxiety and depression levels in patients with long-COVID-19 syndrome: a six-month follow-up study. Diseases. (2024) 12:21. doi: 10.3390/diseases12010021, PMID: 38248372 PMC10814582

[15] Zang C Hou Y Schenck EJ Xu Z Zhang Y Xu J. Identification of risk factors of long COVID and predictive modeling in the RECOVER EHR cohorts. Commun Med (Lond). (2024) 4:130. doi: 10.1038/s43856-024-00549-0, PMID: 38992068 PMC11239808

[16] Sürme Y Özmen N Ertürk Arik B. Fear of COVID-19 and related factors in emergency department patients. Int J Ment Health Addict. (2023) 21:28–36. doi: 10.1007/s11469-021-00575-2, PMID: 34220384 PMC8241404

[17] Soomägi A Meister T Vorobjov S Suija K Kalda R Uusküla A. Fear of COVID-19 among patients with prior SARS-CoV-2 infection: a cross-sectional study in Estonian family practices. Eur J Gen Pract. (2023) 29:2195163. doi: 10.1080/13814788.2023.2195163, PMID: 37259825 PMC10249452

[18] Hansson M Chotai J Nordstöm A Bodlund O. Comparison of two self-rating scales to detect depression: HADS and PHQ-9. Br J Gen Pract. (2009) 59:e283–8. doi: 10.3399/bjgp09X454070, PMID: 19761655 PMC2734374

[19] Varma P Junge M Meaklim H Jackson ML. Younger people are more vulnerable to stress, anxiety and depression during COVID-19 pandemic: a global cross-sectional survey. Prog Neuropsychopharmacol Biol Psychiatry. (2021) 109:110236. doi: 10.1016/j.pnpbp.2020.110236, PMID: 33373680 PMC7834119

[20] Serpytis P Navickas P Lukaviciute L Navickas A Aranauskas R Serpytis R. Gender-based differences in anxiety and depression following acute myocardial infarction. Arq Bras Cardiol. (2018) 111:676–83. doi: 10.5935/abc.20180161, PMID: 30156607 PMC6248233

[21] Sandanger I Nygård JF Sørensen T Moum T. Is women’s mental health more susceptible than men’s to the influence of surrounding stress? Soc Psychiatry Psychiatr Epidemiol. (2004) 39:177–84. doi: 10.1007/s00127-004-0728-6, PMID: 14999449

[22] Egger M Vogelgesang L Reitelbach J Bergmann J Müller F Jahn K. Severe post-COVID-19 condition after mild infection: physical and mental health eight months post infection: a cross-sectional study. Int J Environ Res Public Health. (2023) 21:21. doi: 10.3390/ijerph21010021, PMID: 38248486 PMC10815598

[23] Matsumoto K Hamatani S Shimizu E Käll A Andersson G. Impact of post-COVID conditions on mental health: a cross-sectional study in Japan and Sweden [published correction appears in BMC Psychiatry. BMC Psychiatry. (2022) 22:237. doi: 10.1186/s12888-022-03874-7, PMID: 35379224 PMC8977559

[24] Azizi A Achak D Saad E Hilali A Youlyouz-Marfak I Marfak A. Post-COVID-19 mental health and its associated factors at 3-months after discharge: a case-control study. Clin Epidemiol Glob Health. (2022) 17:101141. doi: 10.1016/j.cegh.2022.101141, PMID: 36119409 PMC9465475

[25] Beauchamp M Kirkwood R Duong M Ho T Raina P Kruisselbrink R. Long-term functional limitations and predictors of recovery after COVID-19: a multicenter prospective cohort study. Am J Med. (2024) 137:990–1000. doi: 10.1016/j.amjmed.2024.06.005, PMID: 38878946

[26] Navas-Otero A Calvache-Mateo A Calles-Plata I Valenza-Peña G Hernández-Hernández S Ortiz-Rubio A. A lifestyle adjustments program in long COVID-19 improves symptomatic severity and quality of life. A randomized control trial. Patient Educ Couns. (2024) 122:108180. doi: 10.1016/j.pec.2024.108180, PMID: 38330704

[27] Schramm SH Moebus S Lehmann N Galli U Obermann M Bock E. The association between stress and headache: a longitudinal population-based study. Cephalalgia. (2015) 35:853–63. doi: 10.1177/0333102414563087, PMID: 25480807

[28] Segerstrom SC Miller GE. Psychological stress and the human immune system: a meta-analytic study of 30 years of inquiry. Psychol Bull. (2004) 130:601–30. doi: 10.1037/0033-2909.130.4.601, PMID: 15250815 PMC1361287

[29] Gold SM Köhler-Forsberg O Moss-Morris R Mehnert A Miranda JJ Bullinger M. Comorbid depression in medical diseases. Nat Rev Dis Primers. (2020) 6:69. doi: 10.1038/s41572-020-0200-2, PMID: 32820163

