# Supplementary material for: Anxiety, depression, and fear after coronavirus disease 2019 infection and their association with long coronavirus disease symptoms
Source: Front Psychiatry. 2025 Sep 10;16:1672447. doi: 10.3389/fpsyt.2025.1672447 (PMC12457336; doi:10.3389/fpsyt.2025.1672447)
Supplement: Supplementary file 1 [file DataSheet1.pdf]

Supplementary Figure 1

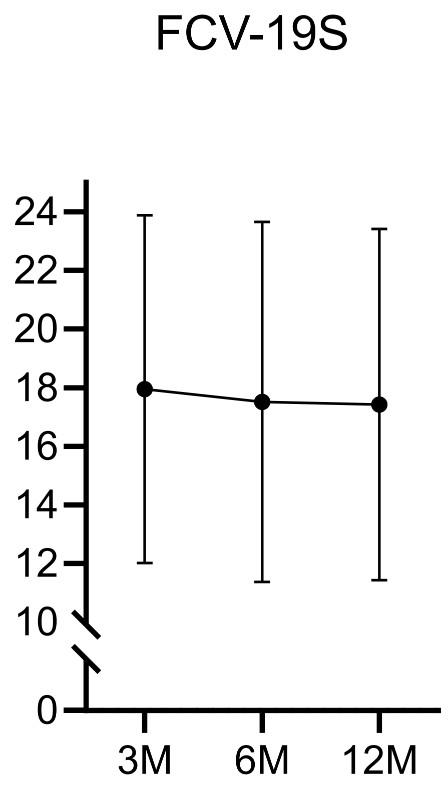

Supplementary Figure 2

A

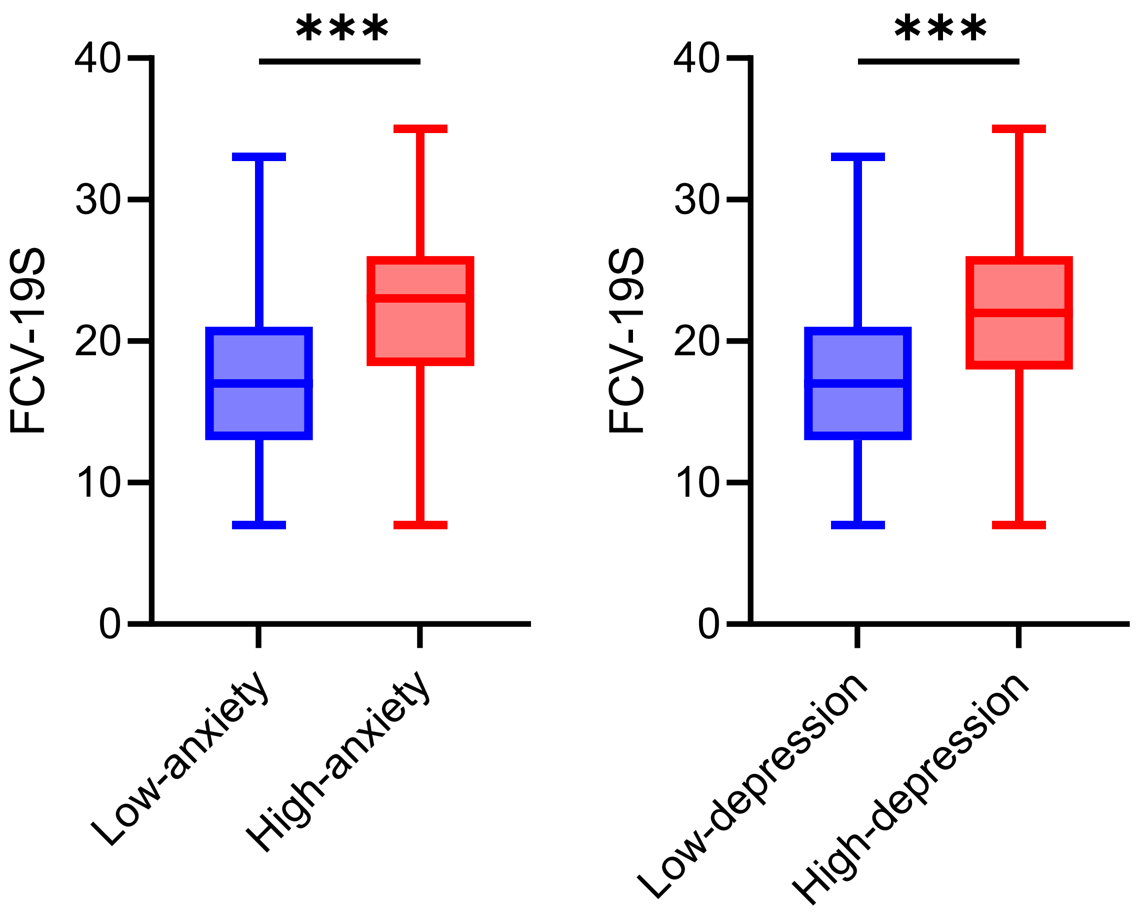

B

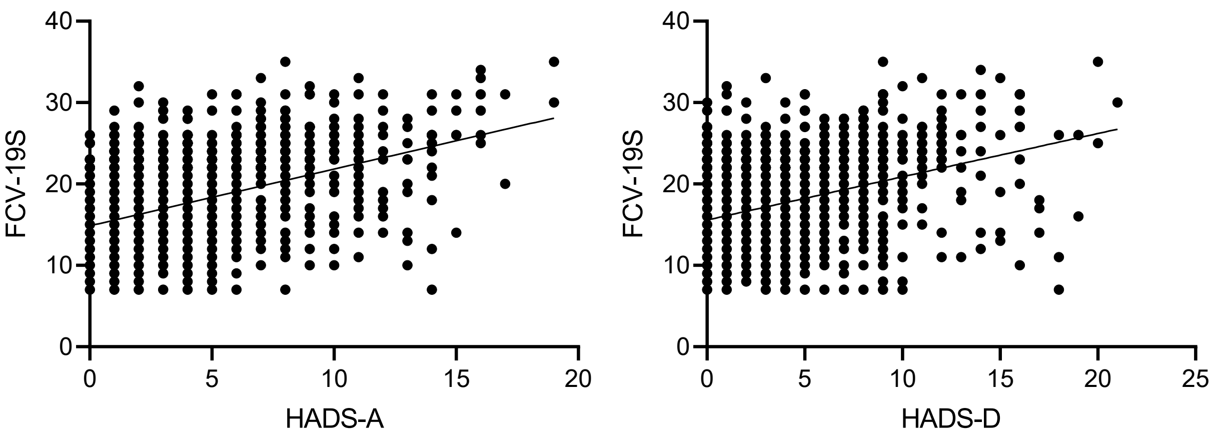

R = 0.45, p < 0.0001

R = 0.38, p < 0.0001

Supplementary Figure 3

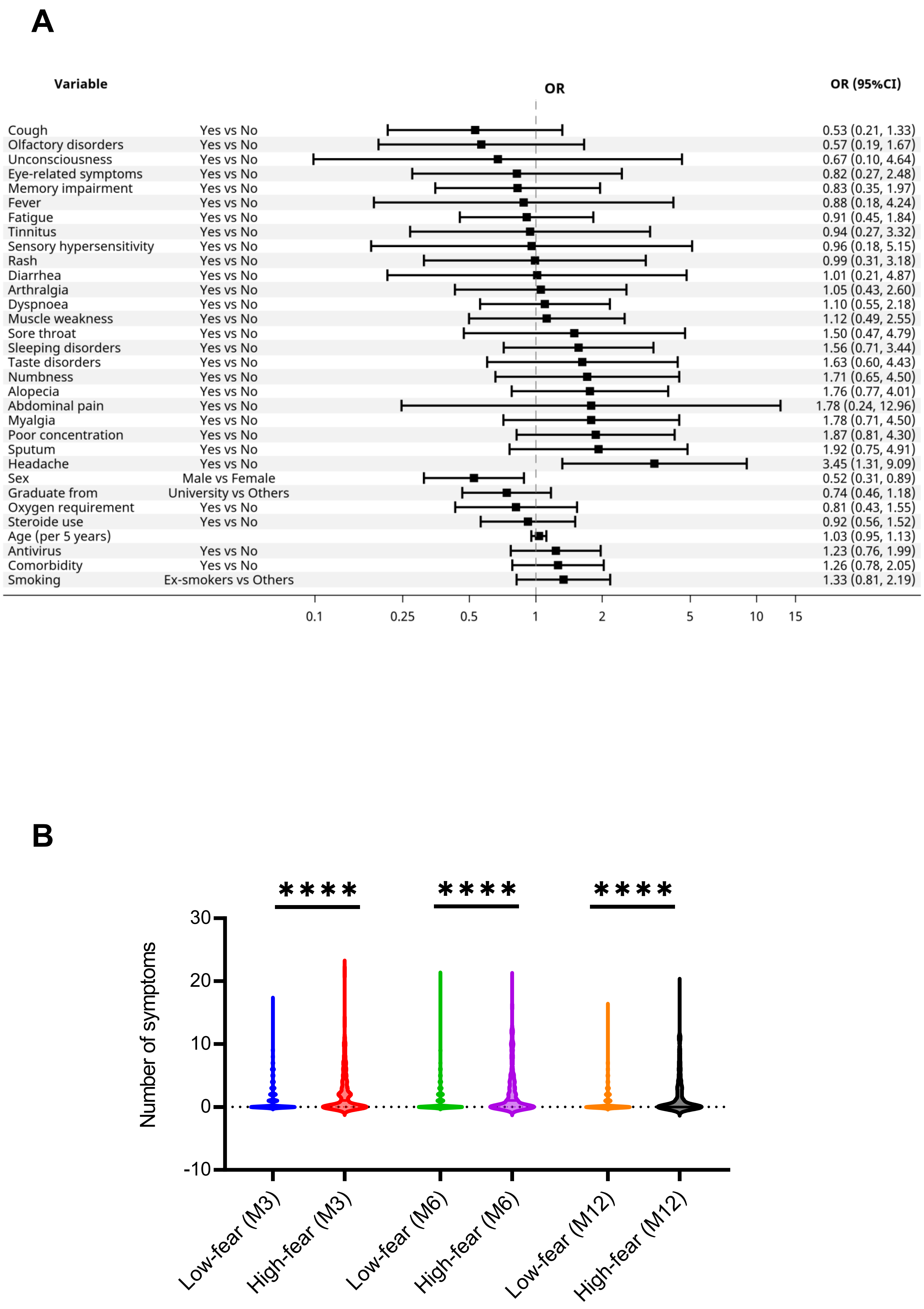

Supplementary Table 1. Baseline Characteristics of the Study Population Recovering from COVID-19

Characteristics	All patients
Age, years	56.1 ± 16.8
Gender, male	679 (63.7)
BMI	24.2 ± 4.1
Smoking status, ex-smoker	374 (35.1)
Oxygen requirement	343 (32.2)
Incubation (including ECMO)	47 (4.4)
Comorbidities	
Hypertension	344 (32.3)
Diabetes mellitus	178 (16.7)
Cardiovascular disease	68 (6.3)
COPD	32 (3.0)
Asthma	55 (5.2)
Hyperuricemia	108 (10.1)
Chronic liver disease	35 (3.3)
Chronic kidney disease	46 (4.3)

Psychiatric disorders	14 (1.3)
Drugs	
Corticosteroids	328 (30.8)
Antivirals	355 (33.3)
QOL score	
HADS-A	4.4 ± 3.9
HADS-D	4.5 ± 4.2
FCV-19S	17.9 ± 5.9

Note. BMI; Body Mass Index. COPD; Chronic Obstructive Pulmonary Disease. ECMO; Extracorporeal Membrane Oxygenation. FCV-19S; Fear of COVID-19 Scale. HADS-A; Hospital Anxiety and Depression Scale-Anxiety subscale. HADS-D; Hospital Anxiety and Depression Scale-Depression subscale. IQR; Interquartile Range. QOL; Quality of Life. Values are presented as mean ± standard deviation (SD) for continuous variables and count (percentage) for categorical variables.

Supplementary Table 2. Comparison of Baseline Characteristics by Anxiety Status (HADS-A)

	M3			M6			M12		
	Low-anxiety n=807	High-anxiety n=203	p value	Low-anxiety n=736	High-anxiety n=176	p value	Low-anxiety n=591	High-anxiety n=131	p value
Age, years	56.6 ± 16.5	52.3 ± 17.3	0.0012*	57.4 ± 16.3	52.6 ± 17.0	0.00050*	59.6 ± 15.6	53.0 ± 16.7	<.0001*
Sex, male	527 (65.3)	113 (55.7)	0.011*	471 (64.0)	102 (58.0)	0.14	374 (63.3)	72 (55.0)	0.078
BMI	24.3 ± 4.3	23.7 ± 5.2	0.19	24.4 ± 3.9	23.9 ± 0.3	0.18	24.1 ± 3.7	24.4 ± 4.6	0.41
Smoking status, ex-smoker	279 (42.2)	72 (42.6)	0.93	251 (41.8)	63 (41.7)	0.98	208 (42.3)	50 (44.3)	0.70
Oxygen requirement	268 (34.1)	57 (28.5)	0.13	240 (33.4)	44 (25.6)	0.047*	207 (35.9)	34 (26.6)	0.043*
Intubation (including ECMO)	36 (4.6)	10 (5.0)	0.78	36 (5.0)	4 (2.3)	0.15	34 (5.9)	3 (2.3)	0.13
Comorbidities									
Hypertension	256 (31.9)	61 (30.4)	0.67	242 (33.0)	53 (30.5)	0.52	222 (37.8)	38 (29.2)	0.067
Diabetes mellitus	134 (16.8)	28 (14.0)	0.34	124 (17.0)	28 (16.0)	0.77	108 (18.5)	16 (12.3)	0.092
Cardiovascular disease	50 (6.3)	12 (6.0)	0.89	50 (6.8)	8 (4.6)	0.28	42 (7.2)	6 (4.7)	0.30
COPD	24 (3.0)	5 (2.5)	0.70	22 (3.0)	4 (2.3)	0.62	19 (3.2)	3 (2.3)	0.58
Asthma	39 (4.9)	12 (6.0)	0.54	34 (4.7)	13 (7.5)	0.13	29 (5.0)	11 (8.5)	0.12

Hyperuricemia	83 (10.4)	21 (10.5)	0.96	75 (10.2)	16 (9.2)	0.67	69 (11.8)	12 (9.2)	0.40
Chronic liver disease	27 (3.4)	6 (3.0)	0.80	29 (4.0)	5 (2.9)	0.50	21 (3.6)	6 (4.6)	0.59
Chronic kidney disease	35 (4.4)	9 (4.5)	0.94	32 (4.4)	9 (5.2)	0.64	30 (5.2)	5 (3.8)	0.52
Drugs									
Corticosteroids	260 (32.7)	50 (25.3)	0.044*	240 (32.9)	41 (24.4)	0.032*	208 (35.7)	30 (23.4)	0.0076*
Antivirals	281 (35.2)	57 (28.4)	0.039*	260 (35.6)	47 (27.3)	0.039*	217 (37.2)	35 (26.9)	0.027*

Note. HADS-A: Hospital Anxiety and Depression Scale-Anxiety subscale, BMI: body mass index, COPD; chronic obstructive pulmonary disease, ECMO: extracorporeal membrane oxygenation

High anxiety was defined as HADS-A ≥ 8.

Statistical comparisons were performed **using Student's t-test for continuous variables and the chi-squared test for categorical variables.**

*$p < 0.05$ was considered statistically significant.

Supplementary Table 3. Comparison of Baseline Characteristics by Depression Status (HADS-D)

	M3			M6			M12		
	Low-depression n=774	High-depression n=236	p value	Low-depression n=707	High-depression n=205	p value	Low-depression n=563	High-depression n=159	p value
Age, years	55.7±16.4)	55.6±18.0	0.90	57.1±16.0	54.3±18.0	0.029*	58.7±15.6	57.4±17.4	0.35
Sex, male	500 (64.6)	140 (59.3)	0.14	454 (64.2)	119 (58.1)	0.11	350 (62.2)	96 (60.4)	0.68
BMI	24.4±3.9	23.9±4.7	0.34	24.4±4.0	23.8±4.7	0.078	24.2±3.8	24.1±4.3	0.67
Smoking status, ex-smoker	270 (42.3)	81 (42.2)	0.97	246 (42.1)	68 (41.0)	0.80	206 (43.3)	52 (40.3)	0.55
Oxygen requirement	246 (32.7)	79 (34.1)	0.70	230 (33.4)	54 (26.9)	0.081	188 (34.3)	53 (34.0)	0.94
Intubation (including ECMO)	33 (4.4)	13 (5.6)	0.42	33 (4.8)	7 (3.6)	0.47	30 (5.4)	7 (4.5)	0.65
Comorbidities									
Hypertension	243 (31.6)	74 (31.6)	0.99	228 (32.3)	67 (33.2)	0.83	206 (36.8)	54 (34.2)	0.55

Diabetes mellitus	125 (16.3)	37 (15.8)	0.85	118 (16.8)	34 (16.8)	1.00	97 (17.4)	27 (17.3)	0.98
Cardiovascular disease	48 (6.3)	14 (6.0)	0.90	43 (6.1)	15 (7.4)	0.50	37 (6.6)	11 (7.0)	0.86
COPD	20 (2.6)	9 (3.9)	0.32	25 (3.6)	1 (0.5)	0.023*	19 (3.4)	3 (1.9)	0.34
Asthma	38 (5.0)	13 (5.6)	0.74	34 (4.9)	13 (6.5)	0.36	29 (5.2)	11 (7.1)	0.38
Hyperuricemia	76 (9.9)	28 (12.0)	0.36	70 (10.0)	21 (10.5)	0.84	63 (11.3)	18 (11.5)	0.95
Chronic liver disease	26 (3.4)	7 (3.0)	0.78	26 (3.7)	8 (4.0)	0.87	19 (3.4)	8 (5.1)	0.35
Chronic kidney disease	32 (4.2)	12 (5.2)	0.52	30 (4.3)	1 1(5.5)	0.48	25 (4.5)	10 (6.3)	0.36
Drugs									
Corticosteroids	242 (31.7)	68 (29.6)	0.54	229 (32.7)	52 (26.4)	0.09	196 (35.3)	42 (27.3)	0.063
Antivirals	269 (35.2)	69 (29.5)	0.11	251 (35.8)	56 (27.9)	0.036*	208 (37.3)	44 (28.0)	0.031*

Note. HADS-A: Hospital Anxiety and Depression Scale-Anxiety subscale, BMI: body mass index, COPD; chronic obstructive pulmonary disease, ECMO: extracorporeal membrane oxygenation

depression was defined as HADS-D ≥ 8.

Statistical comparisons were conducted using **Student's *t*-test for continuous variables and the chi-squared test for categorical variables.**

p < 0.05 was considered statistically significant.

Supplementary Table 4 Comparison of Baseline Characteristics by Fear Status (FCV-19S)

	M3			M6			M12		
	Low-fear N=637	High-fear N=348	p value	Low-fear N=736	High-fear N=176	p value	Low-fear N=591	High-fear N=131	p value
Age, years	54.0±0.6	59.2±0.9	<.0001*	55.4±0.7	58.5±1.0	0.0039*	57.0±0.7	60.5±1.0	0.0054*
Gender, male	430 (67.2)	198 (56.9)	0.0013*	198 (32.8)	133 (43.8)	0.0017*	170 (34.9)	107 (45.0)	0.0093*
BMI	24.2±0.2	24.5±0.2	0.24	24.2±0.2	24.6±0.2	0.14	23.9±0.2	24.8±0.3	0.0039*
Smoking status, ex-smoker	218 (40.9)	128 (45.7)	0.19	201 (39.8)	115 (46.2)	0.10	168 (41.3)	91 (45.5)	0.34
Oxygen requirement	184 (29.3)	128 (38.1)	0.0051*	175 (29.5)	111 (37.8)	0.015*	147 (30.6)	86 (37.9)	0.055
Incubation (including ECMO)	23 (3.7)	23 (6.8)	0.027*	24 (4.1)	14 (4.7)	0.73	24 (5.0)	13 (5.7)	0.71
Comorbidities									
Hypertension	178 (28.0)	133 (38.6)	0.0007*	187 (31.1)	115 (33.4)	0.036*	159 (32.7)	96 (40.9)	0.038*
Diabetes mellitus	93 (14.7)	70 (20.4)	0.022*	97 (16.2)	62 (20.7)	0.11	72 (14.9)	48 (20.5)	0.070
Cardiovascular disease	32 (5.1)	27 (7.8)	0.08	35 (5.8)	20 (6.6)	0.66	28 (5.8)	17 (7.2)	0.51
COPD	16 (2.5)	12 (3.5)	0.39	17 (2.8)	12 (4.0)	0.42	15 (3.1)	8 (3.4)	0.82
Asthma	30 (4.7)	19 (5.6)	0.32	27 (4.5)	21 (7.1)	0.12	22 (4.6)	16 (6.9)	0.21
Hyperuricemia	56 (8.8)	42 (12.2)	0.090	58 (9.6)	37 (12.4)	0.21	55 (11.3)	24 (10.3)	0.70
Chronic liver disease	20 (3.2)	13 (3.8)	0.61	20 (3.4)	12 (4.0)	0.70	16 (3.3)	11 (4.7)	0.40
Chronic kidney disease	25 (4.0)	18 (5.3)	0.34	25 (4.2)	17 (5.8)	0.31	26 (5.4)	9 (3.9)	0.46
Drugs									
Corticosteroids	181 (28.6)	119 (35.1)	0.036*	190 (31.7)	116 (38.8)	0.037*	151 (31.1)	93 (40.1)	0.019*
Antivirals	193 (30.4)	132 (38.7)	0.0052*	181 (30.4)	103 (34.7)	0.20	155 (32.2)	76 (32.8)	0.93

Note. FCV-19S; Fear of COVID-19 Scale. BMI; Body Mass Index. COPD; Chronic Obstructive Pulmonary Disease. ECMO; Extracorporeal Membrane Oxygenation. High fear was defined as FCV-19S ≥ 21.

Statistical comparisons were performed using Student's t-test for continuous variables and the chi-square test for categorical variables.

*$p < 0.05$ was considered statistically significant.

Figure Legends

Supplementary Figure 1. Longitudinal changes in FCV-19S scores.

Supplementary Figure 2. Association between the HADS and FCV-19S

(A) Distribution of low and high HADS scores relative to the FCV-19S scores

(B) Correlation analysis between the HADS (HADS-A and HADS-D) and FCV-19S scores

***; P <0.001

HADS: Hospital Anxiety and Depression Scale, FCV-19S: Fear of COVID-19 Scale

Supplementary Figure 3. Association between FCV-19S and Long COVID symptoms.

(A) Specific Long COVID symptoms associated with high FCV-19S scores.

(B) Relationship between FCV-19S scores and the number of Long COVID symptoms reported by participants.

Statistical comparisons were performed using Student's t-test

****; P <0.0001.
